# Dietary Inulin and *Trichuris suis* Infection Promote Beneficial Bacteria Throughout the Porcine Gut

**DOI:** 10.3389/fmicb.2020.00312

**Published:** 2020-03-04

**Authors:** Sophie Stolzenbach, Laura J. Myhill, Lee O’Brien Andersen, Lukasz Krych, Helena Mejer, Andrew R. Williams, Peter Nejsum, C. Rune Stensvold, Dennis S. Nielsen, Stig M. Thamsborg

**Affiliations:** ^1^Department of Veterinary and Animal Sciences, Faculty of Health and Medical Sciences, University of Copenhagen, Frederiksberg, Denmark; ^2^Department of Bacteria, Parasites and Fungi, Statens Serum Institut, Frederiksberg, Denmark; ^3^Department of Food Science, Faculty of Science, University of Copenhagen, Frederiksberg, Denmark; ^4^Department of Clinical Medicine, Aarhus University, Aarhus, Denmark

**Keywords:** gut microbiota, helminth infection, 16s rRNA sequencing, prebiotics, swine

## Abstract

The gut microbiota (GM) displays a profound ability to adapt to extrinsic factors, such as gastrointestinal pathogens and/or dietary alterations. Parasitic worms (helminths) and host-associated GM share a long co-evolutionary relationship, exerting mutually modulatory effects which may impact the health of the host. Moreover, dietary components such as prebiotic fibers (e.g. inulin) are capable of modulating microbiota toward a composition often associated with a healthier gut function. The effect of helminth infection on the host microbiota is still equivocal, and it is also unclear how parasites and prebiotic dietary components interact to influence the microbiota and host health status. Some helminths, such as *Trichuris suis* (porcine whipworm), also exhibit strong immunomodulatory and anti-inflammatory effects. We therefore explored the effects of *T. suis*, alone and in interaction with inulin, both in fecal microbiota during the infection period and luminal microbiota across four intestinal segments at the end of a 4-week infection period. We observed that *T. suis* generally had minimal, but mainly positive, effects on the microbiota. *T. suis* increased the relative abundance of bacterial genera putatively associated with gut health such as *Prevotella*, and decreased bacteria such as Proteobacteria that have been associated with dysbiosis. Interestingly, dietary inulin interacted with *T. suis* to enhance these effects, thereby modulating the microbiota toward a composition associated with reduced inflammation. Our results show that administration of *T. suis* together with the consumption of prebiotic inulin may have the potential to positively affect gut health.

## Introduction

The porcine gastrointestinal tract (GIT) harbors a diverse and dynamic microbial population, which is involved in gut maturation, immune and pathogen barrier function, vitamin synthesis, and metabolization of certain dietary components (Kim and Isaacson, 2015; Blacher et al., 2017; Holman et al., 2017). These effects make the gut microbiota (GM) of major importance to the digestive physiology and overall health of pigs (Pluske et al., 2018).

The GM differs in composition along the GIT depending on the function associated with each individual segment. The main function of the small intestine is enzymatic food digestion and absorption, with the digesta passing in 3–4 h in pigs (Sciascia et al., 2016), making the terminal ileal microbes mainly associated with enzymatic digestion (Quan et al., 2018). In contrast, the cecum and colon act as the main bioreactors of the GIT, where the digesta is retained for 1–2 days (Sciascia et al., 2016). The cecum exhibits diverse multi-metabolic capabilities, and genes associated with protein metabolism, vitamin metabolism, and polyketide metabolism are up-regulated (Quan et al., 2018). In contrast, the colon is mainly associated with carbohydrate fermentation, and increases the nutrient availability to the host through fermentation of otherwise indigestible polysaccharides, such as dietary fiber (Richards et al., 2005; Quan et al., 2018). The fermentation results in the production of short chain fatty acids (SCFAs) and other metabolites essential for maintaining the gut epithelium (Crespo-Piazuelo et al., 2018). The production of SCFA can be up- or down-regulated by altering the diet, e.g. through inclusion of prebiotics such as inulin, which will increase SCFA production.

Inulin is a polysaccharide, often commercially produced from chicory roots, which passes largely undigested through the small intestine, and is fermented by saccharolytic bacteria in the large intestine (Metzler-Zebeli et al., 2017). Inulin is regarded as a prebiotic due to the fermentation, which in humans confers health benefits from bacteria such as *Faecalibacterium prausnitzii*, some *Roseburia* spp. and *Eubacterium rectale* which produce butyrate through metabolic cross-feeding (De Vuyst and Leroy, 2011; Poeker et al., 2018). These bacteria can be regarded as beneficial bacteria due to their SCFA producing properties, and they may protect the host from mucosal inflammation (Kostic et al., 2014). However, the effects of dietary inulin in pigs are less well-defined but studies indicate that inulin provides protection against pathogens, improves growth and feed efficiency and intestinal microbiota modulation (Grela et al., 2016; Metzler-Zebeli et al., 2017; McCormack et al., 2019). Inulin increases bacterial diversity and relative abundance of Clostridiaceae, and lowers the relative abundances of *Escherichia*, indicating a potential beneficial effect (Sattler et al., 2014). Additionally, dietary inulin selectively stimulates growth of bifidobacteria producing acetate and lactate (Patterson et al., 2010).

Recent studies have shown that parasites of both animals and humans can modulate GM composition (Jenkins et al., 2017; Peachy et al., 2017; Leung et al., 2018). Studies have indicated a reduction in the abundance of carbohydrate-utilizing bacteria in the proximal colon of pigs at days 21 and 53 after experimental infection with *Trichuris suis*, the porcine whipworm, which persisted at least short-term after spontaneous worm expulsion due to acquired immunity (Kringel and Roepstorff, 2006; Li et al., 2012; Wu et al., 2012). Using *Trichuris muris*-infected mice others have shown that major changes in GM take place after day 20 post infection (p.i.) with a decrease in fecal and cecal bacterial diversity (Holm et al., 2015; Houlden et al., 2015). Moreover, *Ascaris suum*, the porcine roundworm, decreased microbial diversity while the abundance of the genera *Prevotella* and *Faecalibacterium* was significantly increased, with a reduction in carbohydrate metabolism in the proximal colon (Wang et al., 2019). Furthermore, *A. suum* is known to release antimicrobial factors which can directly affect bacterial growth in the host gut (Midha et al., 2019). Thus, it is clear that there is a direct link between gastrointestinal nematodes and the host gut microbial environment.

Since many parasites reside in the environmental niche in which microbial fermentation of dietary products (the cecum/colon) takes place, the diet may profoundly impact parasite populations. Dietary inclusion of fermentable carbohydrates thus decreased fecal egg counts, female fecundity and size, and up to 97% reduction in worm burden of another intestinal helminth, *Oesophagostomum dentatum* (porcine nodular worm) (Petkevicius et al., 2001, 2003). In accordance, cecal infusion of SCFAs and lactic acid at day 7 p.i. reduced *O. dentatum* fecal egg count and worm recovery by 98 and 92%, respectively (Petkevicius et al., 2004). Similar effects of dietary inulin have been observed with *T. suis* (Petkevicius et al., 2006). However, other studies have shown either no effect on *T. suis* establishment, but a reduced size and an earlier than usual expulsion of the worms (Thomsen et al., 2005), or even an increased establishment of *T. suis* in pigs fed inulin-rich chicory roots (Jensen et al., 2011).

We recently showed that *T. suis* and inulin synergistically enhance mucosal anti-inflammatory immune responses through suppression of pro-inflammatory genes such as *INFG* and *CXCL9* (Myhill et al., 2018). The aim of the present study was therefore to elucidate effects of the interaction between dietary inulin and parasite infection on the host fecal microbiota over 4 weeks and host luminal microbiota composition along the porcine GIT at necropsy.

## Materials and Methods

### Animals and Study Design

A more detailed description of the study design can be found in Myhill et al. (2018). Briefly, 34 crossbred pigs (Yorkshire × Landrace) were purchased from a commercial farm with a history of absence of nematodes, and all pigs were confirmed parasite-naïve by fecal nematode egg counts and serology upon arrival. These 8-weeks old pigs were randomly allocated into three experimental groups and a control group after stratification for sex and bodyweight (BW), following a two-factorial study design (diet and infection; Supplementary Figure S1A): the *T. suis* group (Ts) (*n* = 9) was orally inoculated with 10,000 embryonated *T. suis* eggs and fed a standard diet; the Inulin + *T. suis* group (I + Ts) (*n* = 9) was also inoculated with *T. suis* eggs, but the diet was modified by supplementation with 10% (w/w) long-chain purified chicory inulin (OraftiHP, Beneo, Belgium), with the same level of energy and protein as the standard diet (Supplementary Table S1); the inulin group (I) (*n* = 8) remained uninfected and was fed the inulin-supplemented diet, while the control group (C) (*n* = 8) served as controls and remained uninfected on the standard diet. All groups were given their respective diets at arrival, and pigs were inoculated with *T. suis* 2 weeks after arrival (Supplementary Figure S1B). Pigs were sacrificed by stunning with captive bolt, followed by exsanguination on day 28 p.i. and enumeration of immature *T. suis* (Myhill et al., 2018).

During the 6-week experimental study period, the pigs were housed in solid concrete-floored pens with feed provided twice daily and water available *ad libitum*. Welfare checks were performed daily, and fecal consistency and BW were recorded weekly. Three pigs were excluded during the study period due to *Lawsonia*-enteritis. The three pigs originated from Groups C and I + Ts (both euthanized day 7 p.i.) and Group I (euthanized day 14 p.i.).

The study was approved by the Danish Animal Experimentation Inspectorate (License No.: 2015-15-0201-00760), and performed at the Experimental Animal Unit, University of Copenhagen according to FELASA guidelines and recommendations.

### Sampling and DNA Extraction

Rectal fecal samples from each pig were collected at the farm of origin (day-18 p.i.) and in our stables on days 0, 14, and 28 p.i. and immediately cooled down on ice; individual 0.25 g subsamples were stored at −80°C. At day 28 p.i., 0.25 g subsamples of thoroughly homogenized digesta from the ileum (taken 10 cm oral to the ileo–cecal junction), cecum (blind end), proximal (20 cm aboral from the ileo–cecal junction), and distal colon (midway between cecum and rectum) were collected from each pig and kept on ice before transfer to −80°C within 1 h. All samples collected during the study were subjected to DNA extraction using Mobio PowerSoil kit (Mobio Laboratories, CA, United States), following the manufacturer’s protocol. The extracted DNA was stored at −20°C until further analysis. Samples from the proximal colon were also investigated by gas chromatography for SCFA concentrations, as described by Myhill et al. (2018).

### Library Preparation and 16S rRNA Amplicon Sequencing

A total of 256 samples were subjected to 16S rRNA gene amplicon sequencing. The preparation of the library consisted of three steps: (1) An initial 20-cycle polymerase chain reaction (PCR), which targeted the V3–V4 region of the 16s rRNA gene, (2) a second 20-cycle PCR, which incorporated specific primers with adaptors and indexes in the amplicons, and (3) magnetic beads-based clean-up and normalization followed by pooling and sequencing using a MiSeq (Illumina, San Diego, CA, United States) at Statens Serum Institut (Copenhagen, Denmark). A thorough explanation of the three steps prior to sequencing can be viewed in Myhill et al. (2018).

Prior to the initial PCR, DNA concentration was measured for each sample using Nanodrop ND-1000 Spectrophotometer (NanoDrop Technologies, DE, United States) and normalized to 10 ng/μL. As all 256 samples were run in a single sequencing step, the primers used for the initial PCR step had incorporated inserts of 0–19 nucleotides length into the primers 388F (5′-ACTCCTAYGGGRBGCASCAG-3′) and 588R (5′-AGCGTGGACTACNNGGGTATCTAAT-3′), which resulted in 16 primer combinations (Supplementary Table S2) and an amplicon of approximately 420 nucleotides. The inserted nucleotides forced a 1:1:1:1 ratio of A:T:C:G for the first 20 sequencing cycles, which ensured an enhanced complexity in line with the principles of phased amplicon sequencing (Wu et al., 2015).

### Sequence Mapping

In order to determine the bacterial composition only high-quality reads were mapped using the BION v. 16.03 package (Danish Genome Institute, Denmark) (McDonald et al., 2016). The workflow consisted of several steps. (1) 1st Quality filtering; filtering of the raw reads by removing primer regions and trimming the ends for low base quality using the sliding-window method (quality > 99% for 14 of 15 and 28 of 30 in the 5′-end and 3′-end, respectively). (2) Sequence joining; forward and reverse reads were joined, if a string of at least 18 nucleotides were at least 90% similar. If this was not possible, they were placed end-to-end and the break-point/gap-point was noted. (3) 2nd Quality filtering; joined reads shorter than 250 nucleotides or sequences with 95% of the nucleotides below a quality of 99% were removed. (4) Chimera checking; sequences were clustered by 96% similarity, and tested for chimeras. (5) Mapping sequences to reference databases through a k-mer-based software mapping to species level; the remaining high-quality non-chimera sequences were divided into 8-mers and merged based on 96% 8-mer similarities (not full-length sequence similarity). The resulting 8-mer lists were matched against an amplicon-targeted subset of the Ribosomal Database Project (RDP), which had at least genus and species name. The 8-mers required a minimum of 60% of the 8-mers to be similar to the RDP reference sequence. (6) Showing mapped/identified species in a table; abundance tables were generated for all taxonomic levels (phylum to species), where only sequences with a 8-mer favorite similarity of 85% were listed. The reads were scaled to 100,000 reads per sample. Generated data sets are available at the European Nucleotide Archive (ENA) under the accession number PRJEB29079.

### Analyses of Alpha- and Beta Diversity

Alpha- and beta diversity analyses were performed using QIIME (v1.9.1). Alpha diversity measures for an observed species (96% oligo similarity) were computed for rarefied abundance tables (90,000 reads per sample) using the alpha rarefaction workflow followed by the “compare alpha diversities” script with non-parametric *t*-test (QIIME v1.9.1). Non-phylogenetic principle coordinate analysis (PCoA) plots were generated based on 10 distance matrices using 10 subsampled abundance tables. The number of sequences taken for each jack-knifed subset was set to 90% of the sequence number within all samples (100,000 reads/sample). Sorensen-Dice (S-D; presence/absence of species) and Bray Curtis (BC; abundance of present species) distance matrices were calculated on rarefied (90,000 reads per sample) abundance tables, and tested for separation between groups at each time point and by each gut segment using Analysis of Similarities (ANOSIM) and Permutational Multivariate Analysis of Variance (PERMANOVA) with 999 permutations (PERMANOVA results are only shown in Supplementary Table S3). The relative distribution of registered phyla and families was calculated based on the normalized abundance table, and summarized at phylum and family level abundance tables.

### Analysis of Composition of Microbes

To analyze differences in bacterial taxa between the four groups, Analysis of Composition of Microbes (ANCOM) was used. The analysis was performed in Rstudio (v1.1.383) with a significance level of 0.05 and correction for multiple testing adjustment set at multcorr = 2. The four groups were compared with each other using ANCOM for each time point (fecal microbiota: days −18, 0, 14, and 28 p.i.) and for each gut segment at day 28 (ileum, cecum, proximal, and distal colon).

## Results

### Dietary Inulin Did Not Affect Worm Establishment

Infection established in all inoculated pigs. Groups Ts and I + Ts had mean worm burdens (all worms were immature) of 4,352 ± 2,079 and 3,838 ± 1,020 (±SD), respectively (*p* > 0.05) (Myhill et al., 2018), indicating no difference in worm establishment at day 28 p.i. irrespective of dietary inulin supplementation.

### Global Sequencing Data

A total of 23,275,094 reads were obtained for 254 samples from four time points and four distinct gut segments, with an average of 91,634 reads per sample. After data trimming, quality and chimera filtering, 5,086,207 high-quality sequences were acquired with an average of 20,024 sequences per sample (range 10,417–33,789) before scaling to 100,000 sequences/sample for normalized scores. The sequences were annotated to 666 independent species belonging to 355 genera, 160 families, 59 orders, 41 classes, and 19 phyla. Twelve samples with less than 10,000 sequences were removed from the data set prior to further analysis.

### Alpha Diversity Was Unaffected by *T. suis* Infection or Inulin

No significant differences were found in any of the alpha diversity indices (Shannon, chao1, and number of observed species) over time for any of the four groups (*p* > 0.05), and likewise no differences were observed between the groups for any of the gut segments (*p* > 0.05). There were no significant differences between mean numbers of observed species in ileum and the segments of the large intestine (LI, combining cecum, proximal, and distal colon) in any of the groups [ileum: 114 ± 24 (±SD); LI: 118 ± 26] (Supplementary Figure S2).

**FIGURE 1:**
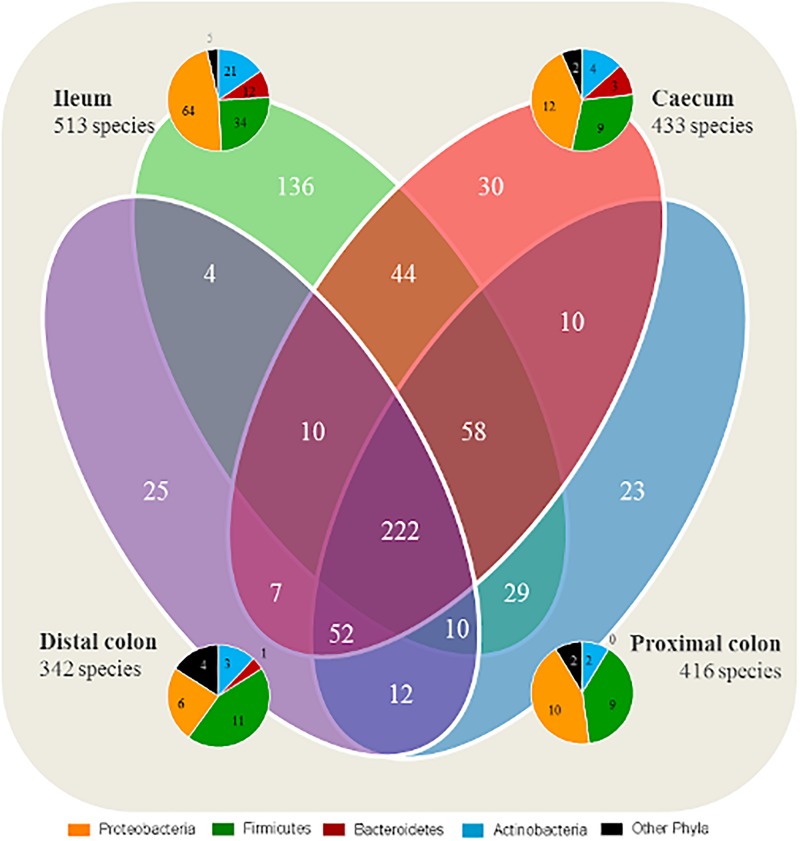
Ileum has a larger quantity of species exclusive to the segment compared with the large intestine. Number of shared microbial species between the intestinal segments, and species exclusive to each segment. All four groups are included for each segment. The taxonomy of exclusive species found in each intestinal segment (ileum, cecum, proximal, and distal colon) are represented by pie charts.

A total of 222 species were identified as shared between all intestinal segments irrespective of group (Figure 1). A total of 136 species were found to be exclusive to the ileum, whereas 23–30 species were exclusive to each segment of the LI. In general, the species found exclusively in a segment were mainly representing members of the Proteobacteria and Firmicutes phyla.

### Inulin and *T. suis* Changed the Fecal Microbiota Over Time

**FIGURE 2:**
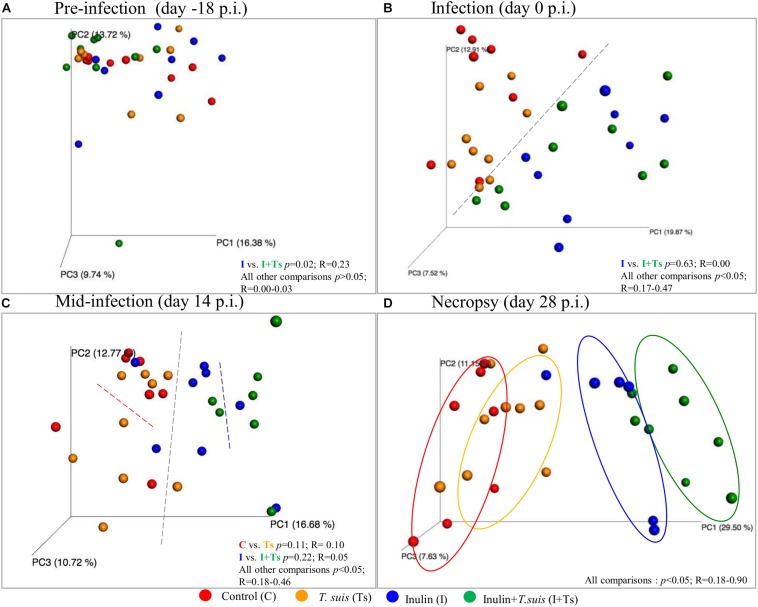
Inulin and *Trichuris suis* alters the fecal microbiota over time into four distinct communities. Principle coordinates analysis (PCoA) plots based on Sorensen–Dice distance matrix of fecal microbiota over the course of *T. suis* infection and inulin supplementation: **(A)** pre-infection [day-18 post-infection (p.i.)]; **(B)** infection (day 0 p.i.); **(C)** mid-infection (day 14 p.i.); and **(D)** necropsy (day 28 p.i.). Respective ANOSIM *R*-values showing the extent of community variation and statistical significance indicated between any two groups at each time point.

Fecal microbiota compositional differences between groups were observed over the course of the experiment. Initially, the four groups were similar in microbiota composition, with the exception of Groups I vs. I + Ts (S-D: *p* = 0.02, *R* = 0.23 and BC: *p* = 0.03, *R* = 0.33) (Supplementary Table S4 and Figure 2A). However, after 14 days of supplementation these groups were similar in composition (S-D: *p* = 0.63, *R* = 0.00), and the four groups separated into two distinct clusters based on dietary supplementation (Figure 2B, S-D and BC: *p* < 0.05); this difference persisted until termination of the experiment at day 28 p.i. After 14 days of infection, there were no significant difference between infected and naïve groups (Figure 2C and Supplementary Table S4). However, after 28 days of infection, four distinct group-dependent clusters had formed (Figure 2D and Supplementary Table S4) with increasingly greater differences in microbial composition, as evidenced by an increasing *R*-value. All groups were significantly different by S-D (Group C vs. Group Ts: *p* = 0.04, *R* = 0.18; all other combinations *p* < 0.005), but infection did not affect the composition measured by abundance (BC, Group C vs. Group Ts: *p* = 0.11, *R* = 0.12; Group I vs. Group I + Ts*: p* = 0.10, *R* = 0.16) (Supplementary Table S4) indicating that the observed differences in the *T. suis* infected groups are mainly driven by low-abundant GM members. Investigating the changes in S-D indices within each group (Supplementary Table S5), it was observed that after initial acclimatization, Group C and Group I remained stable from day 0 to day 28 p.i. with no differences between time points. In Group Ts, there were no changes in the microbial composition within the first 14 days, whereas the overall change from day 0 to day 28 p.i. was significant (S-D: *p* = 0.004, *R* = 0.23). Group I + Ts responded strongly to the two-factor interaction, which resulted in an altered microbial composition between any two time points (S-D: *p* < 0.02, *R* = 0.14–0.43).

### The Luminal Microbiota Along the GIT Were Affected by *T. suis* Infection

**FIGURE 3:**
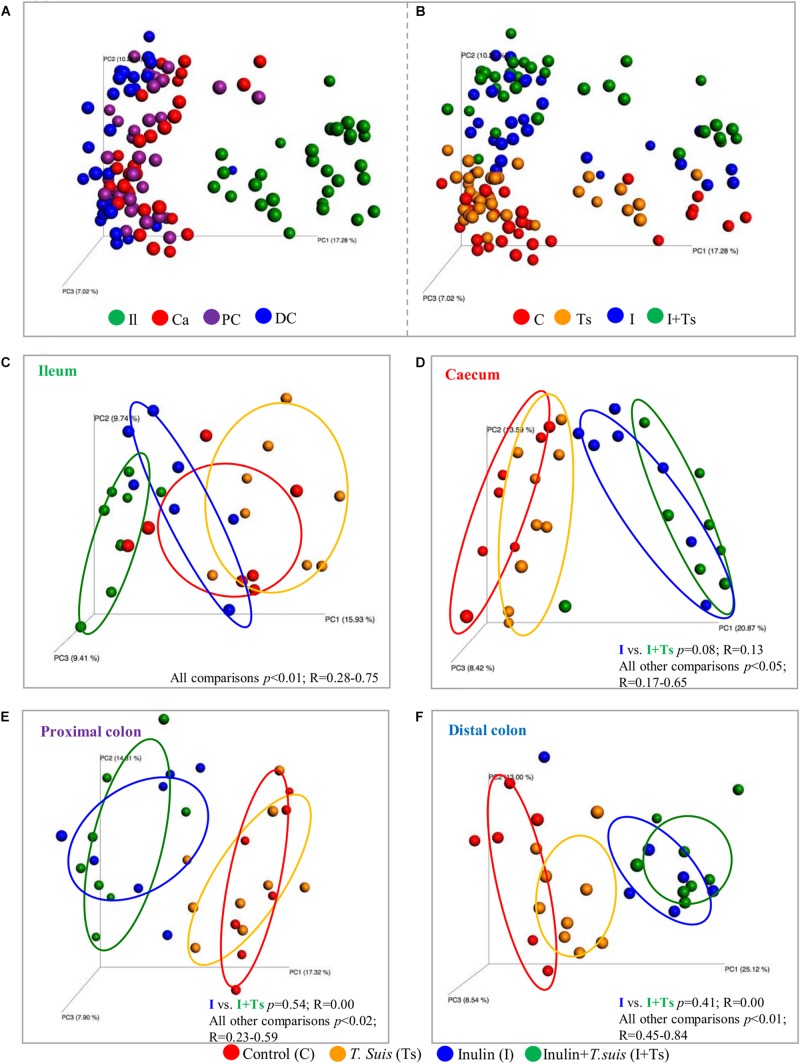
Inulin alters the microbial composition to the greatest extent, but *Trichuris suis* alters the composition in each intestinal segment as well. Principle coordinates analysis (PCoA) plot based on Sorensen–Dice distance matrix comparing the luminal microbiota at day 28 post infection (p.i.) analyzed according to **(A)** intestinal segment and **(B)** experimental group. **(C–F)** Comparison of microbiota within each intestinal segment displays how the four groups clusters within each intestinal segment. **(C)** ileum (Il); **(D)** cecum (Ca); **(E)** proximal colon (PC); and **(F)** distal colon (DC). Respective ANOSIM *R*-values show the extent of community variation and level of statistical significance between any two groups in each location. C, control group; Ts, inoculated with *T. suis*; I, inulin-supplemented diet; I + Ts, inulin-supplemented diet and inoculation with *T. suis*.

As the experimental treatments strongly influenced the fecal microbiota composition at day 28 p.i., we further investigated whether similar effects could be found within the intestinal segments. The LI segments clearly clustered separately from the ileum (S-D *p* ≤ 0.05; BC ≤ 0.05) (Figure 3A and Supplementary Table S6), indicating different microbial composition between these two parts of the intestinal tract. Within the LI, experimental treatment with I + Ts, but not I or Ts alone, resulted in significant differences in S-D indices for cecum vs. distal colon and for proximal vs. distal colon (Supplementary Table S7), indicating that the combined interaction affected the microbial composition to a greater extent in LI compared with single experimental treatments. Similar to Group I + Ts, Group C had significant differences in S-D indices for cecum vs. distal colon and for proximal vs. distal colon (Supplementary Table S7).

In general, inulin supplementation drove the separation between groups for each individual gut segment in contrast to the effect of *T. suis*, which was dependent on location (Figures 3B–F). With regard to the ileal samples, all groups were observed to separate by both unweighted S-D as well as relative abundance-weighted BC indices. In the cecum, proximal and distal colon, significant differences between groups were found for S-D indices, including a separation of Group Ts and Group C; however, there were no significant differences between Groups I + Ts and I (Supplementary Table S6A). Mainly low-abundant species were affected by infection in the cecum and proximal colon, as no separation between Groups C and Ts was observed when BC-based indices were considered (BC: cecum: *p* = 0.13, *R* = 0.09; proximal: *p* = 0.06, *R* = 0.17). However, in the distal colon both high- and low-abundance species were affected in Group Ts (BC: *p* = 0.04, *R* = 0.25). Similar trends were observed for BC indices (Supplementary Table S6B).

### A Strong Interaction Between *T. suis* and Inulin Affects the Fecal Composition of Taxa Over Time

**FIGURE 4:**
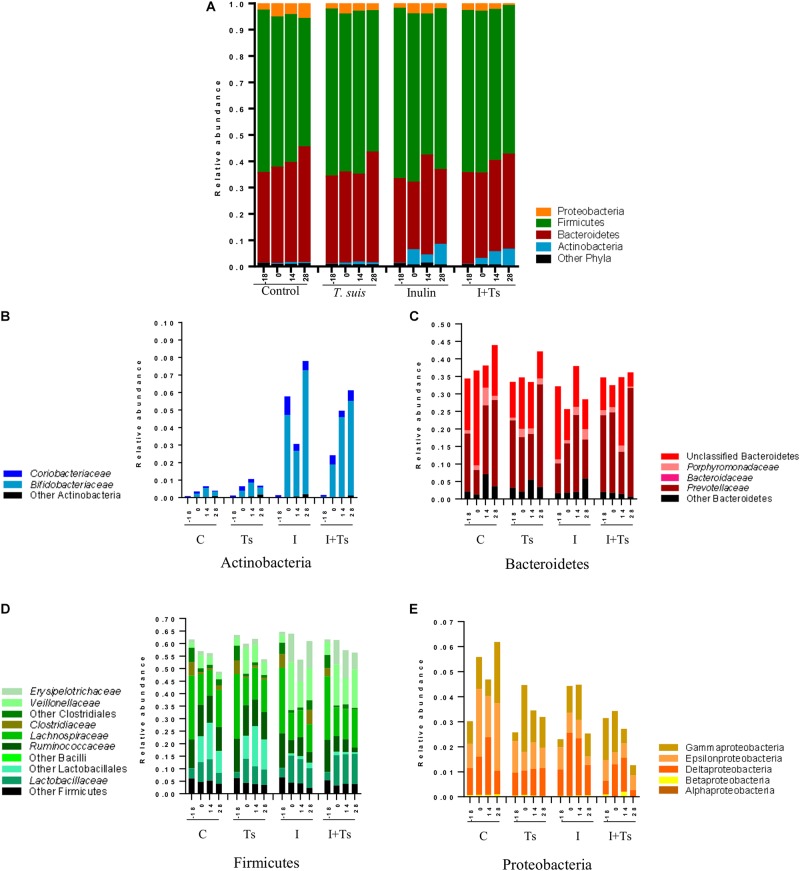
The interaction between inulin and *Trichuris suis* mainly affects Prevotellaceae and Proteobacteria over time. Mean relative abundance of the four main phyla **(A)**, and lower taxonomic levels in each phylum, **(B)** Actinobacteria, **(C)** Bacteroidetes, **(D)** Firmicutes, **(E)** Proteobacteria, for each experimental group over four time points [day-18 post-infection (p.i.; pre-infection); day 0 p.i. (infection); day 14 p.i. (mid-infection); day 28 p.i. (necropsy)] (C, control; Ts, *T. suis*; I, inulin; I + Ts, inulin + *T. suis*).

To examine what drives the separation between groups, the distribution of taxa in feces was analyzed at each time point. The changes caused by experimental treatments were visible both at phylum (Figure 4A) and family/class levels (Figures 4B–E), and can be attributed to specific taxa by ANCOM analysis (Supplementary Figure S3).

**FIGURE 5:**
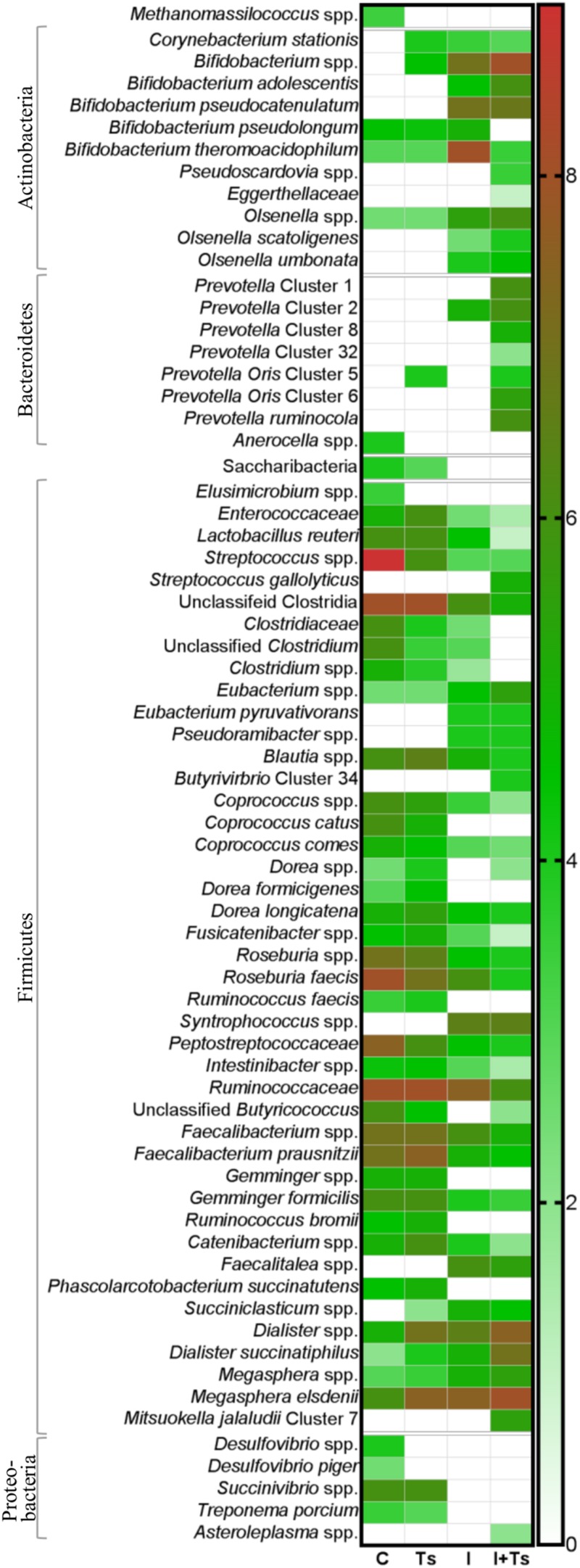
The relative abundance of specific taxa is altered depending on experimental treatment. Heatmap of significantly different log abundances of fecal taxa at day 28 post-infection (p.i.). Only taxa found to be significantly different by ANCOM (*p* < 0.05) between two or more groups are included (C, control; Ts, *Trichuris suis*; I, inulin; I + Ts, inulin + *T. suis*).

For inulin-supplemented groups, the most dominant change was the increase in Actinobacteria due to Bifidobacteriaceae from day 0 to 28 p.i. (Figure 4B). At day 28 p.i., the relative abundance of three species of *Bifidobacterium* and *Olsenella* was significantly higher (Figure 5). Likewise, a higher abundance of certain families within Firmicutes was observed, although the overall abundance of Firmicutes was similar (Figure 4D). Especially Lactobacillaceae, Veillonellaceae, and Erysipelotrichaceae increased noticeably, while Lachnospiraceae, Clostridiaceae, Ruminococcaceae, and other Lactobacillales, such as *Streptococcus*, decreased compared with the control-fed groups at day 28 p.i. (Figure 5). These changes were observed after 14 days of inulin supplementation, and persisted, albeit with some fluctuations, for the duration of the study.

Group Ts did not exhibit changes in the microbial composition to the same extent as groups supplemented with inulin. As seen earlier, the changes were only significant at day 28 p.i., and were mainly attributed to a higher relative abundance of Prevotellaceae (Bacteroidetes), and significant increases in the relative abundance of specific *Prevotella* clusters compared with the other groups (Figures 4C, 5). A slight decrease was observed in the abundance of Proteobacteria at day 28 p.i. compared with earlier time points for Group Ts, and compared with Group C (Figure 4E). For Firmicutes, ANCOM showed an increase in *Dorea formicigenes*, *Dialister* spp., *Succiniclasticum* spp., and *Megasphera elsdenii* and a concurrent decrease in *Streptococcus* spp., several *Clostridium* spp., and *Butyricicoccus* spp. (Figure 5).

The microbial composition was similar for Groups I + Ts and I at days 0 and 14 p.i., but at day 28 p.i. certain taxonomical changes could be observed. The interaction between inulin and *T. suis* showed two main tendencies at day 28 p.i.: a higher abundance of Prevotellaceae, which led to the overall increase in Bacteroidetes similar to the observation for Group Ts (Figure 4C), and a substantial decrease in Proteobacteria, mainly of Succinivibrionaceae, Enterobacteriaceae, and Campylobacteraceae, belonging to Gamma- and Epsilonproteobacteria, respectively (Figures 4E, 5).

Interestingly, the interaction between inulin and *T. suis* lowered the relative abundance of *Bifidobacterium pseudolongum* and concomitantly increased two other Actinobacteria taxa. Prevotellaceae increased, including *P. ruminicola* which was low-abundant or not detected in the remaining three groups (Figure 5). In Group I + Ts, Firmicutes decreased slightly from days 0 to 28 p.i., mainly due to a decrease in *Lactobacillus reuteri*, *Roseburia faecis*, and *F. prausnitzii*, among others, whereas the class of Negativicutes increased due to *Megasphaera* spp. (Figure 5).

In brief, inulin supplementation resulted in a rapid alteration of the microbiota mainly through higher relative abundances in *Bifidobacterium* and Lactobacilliceae and a lower relative abundance of, e.g. Lachnospiraceae, which persisted throughout the study. In contrast, compositional changes induced by *T. suis* alone were not seen until day 28 p.i., when mainly low-abundant species were affected and a lower abundance of Proteobacteria was seen. The interaction between inulin and *T. suis* resulted in a composition very similar to inulin treatment alone until day 28 p.i., where after the interaction resulted in a higher relative abundance of *Bifidobacterium* and Prevotellaceae and a lower abundance of Proteobacteria compared to the individual experimental treatments.

### Inulin and *T. suis* Affected Each GIT Segment Differently

**FIGURE 6:**
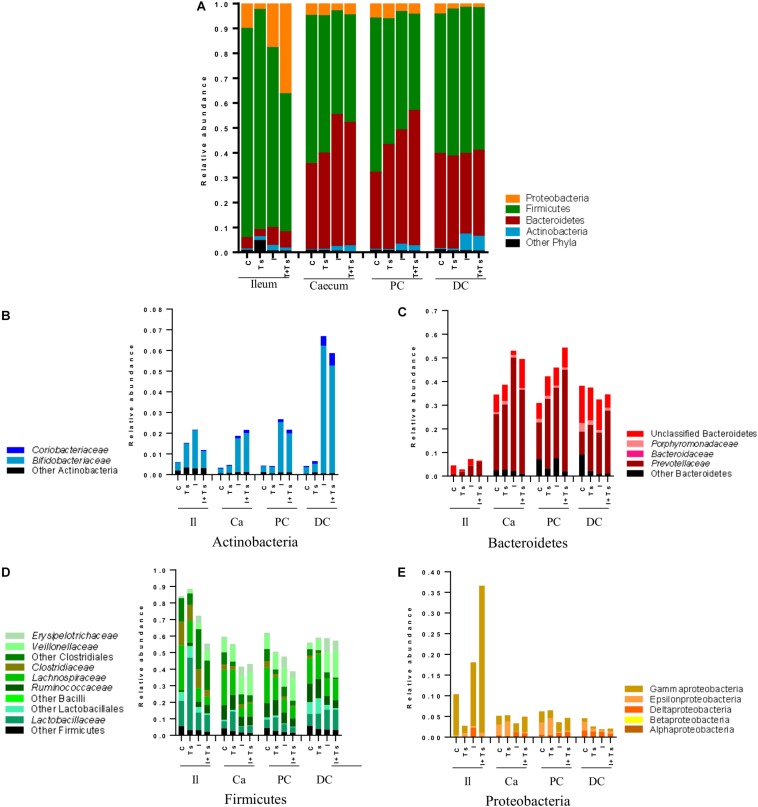
Inulin enhances *Bifidobacterium* in all segments, but the interaction between inulin and *Trichuris suis* alters the taxonomic distribution further throughout the gastrointestinal tract. Mean relative abundance of the four main phyla **(A)**, and lower taxonomic levels in each phylum, **(B)** Actinobacteria, **(C)** Bacteroidetes, **(D)** Firmicutes, and **(E)** Proteobacteria, for each experimental group in four distinct intestinal segments. The increase in “other phyla” observed for ileum belongs to Tenericutes (Mycoplasmataceae) (Il, ileum; Ca, cecum; PC, proximal colon; DC, distal colon; C, control; Ts, *T. suis*; I, inulin; I + Ts, inulin + *T. suis*).

At day 28 p.i., there were clear differences at phylum level between the ileum and LI (Figure 6A). Samples from ileum were dominated by Firmicutes and Proteobacteria. The cecum and proximal colon appeared to have a similar taxonomic profile with approximately the same Firmicutes:Bacteroidetes ratio within each experimental group, and a lower abundance of Proteobacteria compared with the ileum. Likewise, distal colon was dominated by Firmicutes and Bacteroidetes, but a lower abundance of Proteobacteria and a higher abundance of Actinobacteria were observed in the inulin-supplemented groups.

**FIGURE 7:**
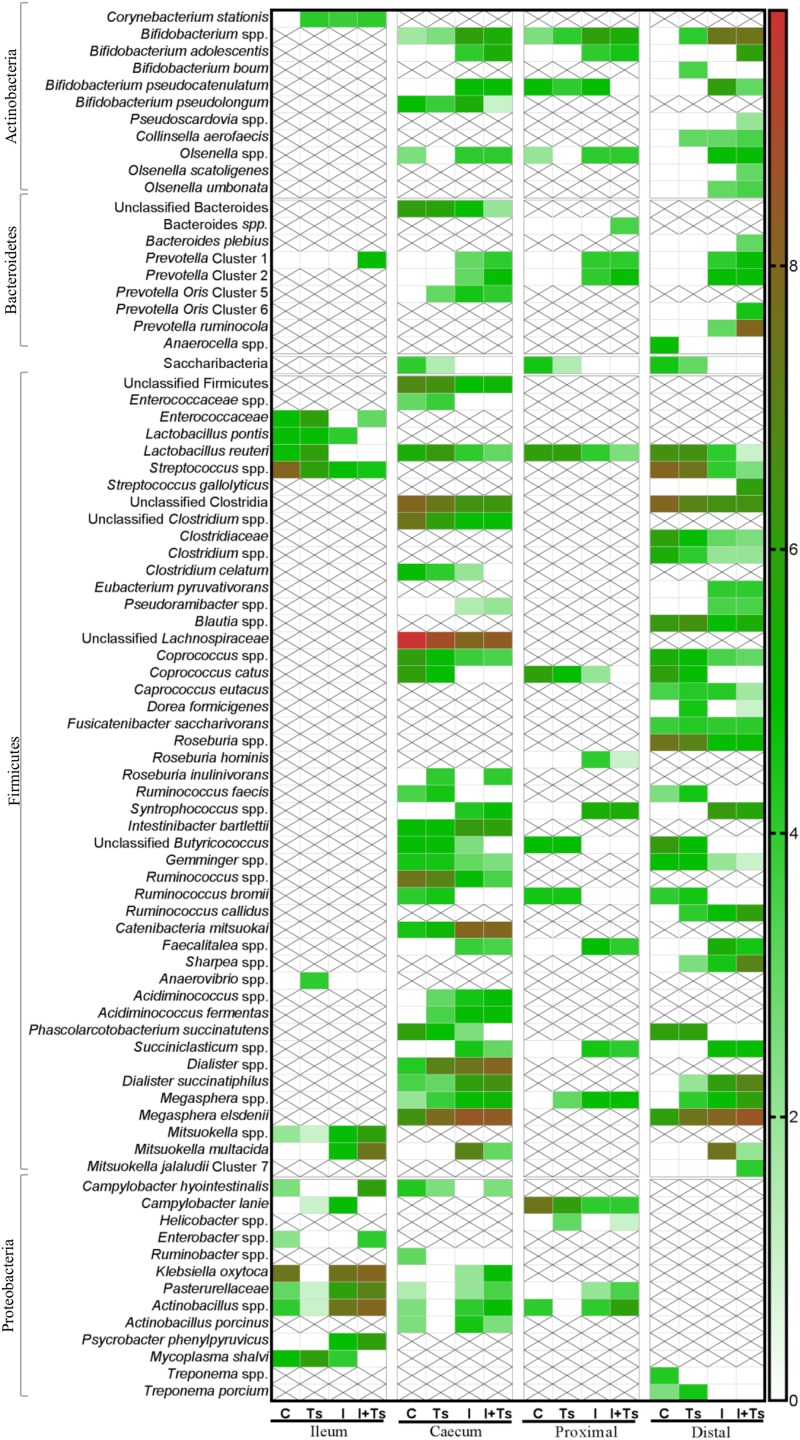
The experimental treatment affected specific taxa more than intestinal segment. Heatmap of significantly different log abundance of luminal taxa at day 28 post-infection (p.i.). Only taxa found to be significantly different by ANCOM (*p* < 0.05) between two or more groups are included. X denotes no difference in abundance in the segment, not non-presence (C, control; Ts, *Trichuris suis*; I, inulin; I + Ts, inulin + *T. suis*).

Between groups, there were apparent differences in the relative abundance of families in each segment. In Group Ts, a strikingly higher relative abundance of Lactobacillaceae was observed in ileum, and this group also exhibited a generally lower relative abundance of Proteobacteria compared with Group C (Figures 6A,E). Group Ts had generally few changes in cecum and proximal colon compared with Group C, although a slight increase in Prevotellaceae (Figure 6C) and a higher relative abundance *Megasphaera* spp., *Roseburia inulinivorans*, *Ruminococcus faecis*, and *L. reuteri* were apparent in cecum. *Coprococcus catus* and *Ruminococcus bromii* were highly abundant in all LI segments for both groups Ts and C compared with inulin-supplemented groups (Figure 7). A decrease in the relative abundance of *Olsenella* spp., Pasteurellaceae and *Actinobacillus* spp. from the ileum to the proximal colon, and a decrease in Campylobacteraceae in the distal colon could be observed for Group Ts.

In all four segments, the relative abundance of *Bifidobacterium* spp. was generally higher for inulin-supplemented groups and tended to increase from the cecum to the distal colon (Figure 6B). At the single species level, *Bifidobacterium adolescentis* decreased dramatically from proximal to distal colon in Group I, and in Group I + Ts *Bifidobacterium pseudocatenulatum* and *B. pseudolongum* decreased from cecum to proximal colon. *Bifidobacterium boum* was only detected in the distal colon for Group Ts (Figure 7).

For the inulin-supplemented groups, a higher relative abundance of Negativicutes and a concomitantly lower relative abundance of Lachnospiraceae was observed in the ileum, and Group I + Ts exhibited a larger proportion of Gammaproteobacteria (mainly Enterobacteriaceae and Pasteurellaceae) (Figures 6D,E).

Likewise, a higher relative abundance of Prevotellaceae could be observed, with the greatest abundance for Group I in the cecum and Group I + Ts in the proximal and distal colon, albeit generally highest in the cecum and proximal colon (Figure 6C). Specific *Prevotella* taxa were affected by segment and group. *Prevotella* Cluster 1 was present in all segments for Group I + Ts, whereas *P. oris* Cluster 5 was only detected in the cecum, being higher in abundance for all experimental groups compared with Group C. For Group I + Ts, the abundance of *P. ruminicola* and *P. oris* Cluster 6 was higher in the distal colon compared with Group I (Figure 7).

The higher abundance of Bacteroidetes concurrently led to a lower abundance of Firmicutes for the cecum and proximal colon (Figures 6C,D, 7) for Groups I and I + Ts, represented mainly by Clostridiaceae, Lachnospiraceae, and Ruminococcaceae, but a higher relative abundance of Erysipelotrichaceae.

For the distal colon, inulin supplementation mainly resulted in a higher relative abundance of Negativicutes, but also a lower relative abundance of Epsilonproteobacteria compared with Groups C and Ts and other segments (Figure 6E).

Overall, the results indicated large differences in microbiota composition between ileum and the large intestine, and likewise a difference in the segments between the experimental groups. For each group specific taxa, e.g. *Bifidobacterium*, were affected differently with specific species found in each group. Additionally, the abundance of these taxa was higher or lower depending on segment.

## Discussion

The current study explored if dietary inulin can be used to modulate the microbiota of pigs toward a composition associated with a “healthy gut” even while exposed to intestinal parasites. The combination of dietary inulin and *T. suis* infection appeared to cooperatively enhance the effects seen in each experimental treatment, resulting in a higher relative abundance of *Prevotella* and *Bifidobacterium*, and a lower relative abundance of Proteobacteria. These alterations, when taken together with an augmented immune response from *T. suis* resulting in increased transcription of mucosal barrier genes and suppressed anti-inflammatory genes, as reported earlier (Myhill et al., 2018), indicate that pigs fed inulin and colonized with *T. suis* exhibit an anti-inflammatory gut environment, which is richer in potentially beneficial microbes.

The higher relative abundance of *Prevotella* and *Bifidobacterium* and the concomitantly lower relative abundance of Firmicutes and Proteobacteria caused by inulin alone were observed early in the experiment and persisted throughout the study, and were likewise observed in the large intestinal segments, especially in the distal colon. The changes related to *T. suis* infection were less obvious and mainly affected low-abundant species. The main findings were a lower relative abundance of Proteobacteria and a higher abundance of SCFA-related taxa, such as *Dialister* and *Megasphaera*. No prominent changes were observed prior to day 28 p.i., which is likely due to the parasite’s life cycle. After hatching of a *T. suis* egg, the larva burrows into the host epithelial layer, limiting the possibility to interact with the host microbiota. At approximately day 16 p.i. the posterior end of the larva protrudes into the lumen with the anterior end embedded in the epithelium, but it does not reach full maturity until days 42–49 p.i. (Beer, 1973; Kringel and Roepstorff, 2006). Microbial alterations due to *T. suis* are thus likely to be transient and may change with time due to worm development, emergence, maturity with egg excretion, and ultimately expulsion due to host immunity.

We observed a higher relative abundance of Prevotellaceae, as well as specific *Prevotella* taxa, in pigs infected with *T. suis.* A similar increase in Bacteroidetes was also seen in earlier studies in pigs infected with *T. suis*, where the relative abundance of *Paraprevotella* increased substantially, while *Desulfovibrio* and Firmicutes decreased at 21 and 53 days p.i. (Li et al., 2012; Wu et al., 2012). Likewise, *Trichuris trichiura*, the human whipworm, induced an increase in the abundance of *Paraprevotella*, indicating similar effects from *Trichuris* infection in humans (Lee et al., 2014). However, a higher abundance of Bacteroidetes was not observed in mice infected with *T. muris.* Instead, higher relative abundances of Firmicutes and Proteobacteria, along with a lower alpha diversity were observed (Holm et al., 2015; Houlden et al., 2015), which suggests that changes in the GM profiles might be specific to different host/helminth models. The host/helminth interaction likewise indicates an evolutionary arms race, as lower infection rates have been observed in mice with *T. muris*-altered microbiota, suggesting the altered host-microbiota would be less favorable for egg hatching and establishment (White et al., 2018). However, at the same time, the helminth itself selects for its own distinct microbiota independent of the host microbiota, which might be linked to survival of the helminth within the host (White et al., 2018). Together, this indicates that *Trichuris* and the host both contribute to the altered host microbiota.

Here, we found increased relative abundance of Prevotellaceae in all experimental groups compared with the controls, but the species affected were different depending on intestinal segment and experimental treatment. The *Prevotella* genus is commensal to the mammalian gut, and is generally associated with a plant-rich diet and fiber degradation. The fermentation leads to increased amounts of SCFA, which interacts with the immune system and has anti-inflammatory effects (Koh et al., 2016; Rivière et al., 2016), suggesting that *Prevotella* is a generally beneficial microbe. *Prevotella* is found in the GIT of humans and pigs and is driving one of the major “enterotypes” of the human GIT (Arumugam et al., 2011; Ley, 2016). This ubiquitous enterotype is linked to a relatively high total SCFA production, indicating a higher fiber utilizing capacity than other enterotypes (Chen et al., 2017). We saw that the *Prevotella* clusters affected were unique to each experimental group. At day 28 p.i. we found a higher relative abundance of *P. ruminicola* in Group I + Ts compared with other groups. *P. ruminicola* is a propionate producer (Hosseini et al., 2011) and was found only in this group, indicating that each experimental treatment can alter the microbiota distinctively. Given the proposed beneficial properties of *Prevotella*, the increased relative abundance in *T. suis*-infected groups, and that unique clusters are affected, indicates that a moderate *T. suis* infection might be beneficial to some aspects of the gut health of the host.

Likewise, we also saw different *Bifidobacterium* species increasing in relative abundance for each experimental group. *Bifidobacterium* are mainly associated with a healthy gut due to their immunomodulatory (Ruiz et al., 2017) and SCFA-producing properties (Rivière et al., 2016), and they are therefore often used as probiotics to boost gastrointestinal health. The relative abundance of *Bifidobacterium* was mainly affected by inulin supplementation, and less so by *T. suis*. However, it was remarkable that the highest relative abundance of *Bifidobacterium* was observed for the interaction between inulin and *T. suis.* This interaction appeared to act additively and increased the genus notably, a situation also seen for *Prevotella* and *Lactobacillus*. The relative abundance of *Lactobacillus* tripled for the interaction between inulin and *T. suis*, whereas *T. suis* or inulin supplementation alone had a lesser effect on the relative abundance of the genus. Similarly to *Prevotella*, *Lactobacillus* is a commensal genus with some strains regarded as probiotic and thus can provide health benefits to their host; e.g. certain *L. reuteri* strains (Mu et al., 2018). We observed a lower abundance of *L. reuteri* for all intestinal segments in inulin-supplemented groups, but a higher abundance of unclassified *Lactobacillus* species and *L. reuteri* in the *T. suis*-infected group. Increased relative abundance of *Lactobacillus* spp. has also been associated with *T. muris* infections (Holm et al., 2015; Houlden et al., 2015; Duque-Correa et al., 2019), and mice were more susceptible to *T. muris* infection when administered with a probiotic *Lactobacillus* strain (Dea-Ayuela et al., 2008). It can therefore be speculated that *T. muris* can actively increase *Lactobacillus* spp. as part of a survival strategy. This is in line with findings suggesting *T. muris* actively modulates bacterial species to aid helminth establishment and/or survival in the host (White et al., 2018).

The interaction between *T. suis* and inulin affected the microbiota to a greater extent than the single experimental treatments by enhancing the effects from both. We observed changes in certain taxa used as biomarkers for intestinal health; e.g. *F. prausnitzii* and *M. elsdenii*, where *M. elsdenii* was markedly elevated in the interaction group. Both bacterial species are commensals of the pig GIT and involved in the production of SCFA, of which butyrate promotes healthy mucosal tissue, and propionate acts as a glucogenic metabolite for the pig. Besides *F. prausnitzii* and *M. elsdenii*, several other species associated with the production of SCFA were identified, e.g. *C. catus* (propionate) (Reichardt et al., 2014), *R. inulinivorans* (butyrate) (Rivière et al., 2016), *Dialister* spp. (propionate), *Eubacterium* spp. (butyrate), *Prevotella* spp. (propionate, acetate), and *Bifidobacterium* spp. (acetate) (Koh et al., 2016). It is well-known that SCFA associated with these bacteria partake in maintaining gut and immune homeostasis in the host (Koh et al., 2016), yet it is difficult to evaluate whether these induced changes have implications for the worms, such as decreased survival, as intestinal infusion of high levels of SCFA may result in worm expulsion (Petkevicius et al., 2004).

A high relative abundance of Proteobacteria is generally regarded as either an indicator of dysbiosis, or as a direct cause of dysbiosis due to the pathogenic nature of some species in the phylum (Shin et al., 2015; Heinritz et al., 2016). Concomitantly with the increase in the beneficial bacterial species, we saw a generally lower abundance of Proteobacteria for both *T. suis* and inulin alone, and interestingly, an even greater reduction for the combined inulin and *T. suis* group, indicating a strong interaction. This decrease was due to a general reduction in unclassified Deltaproteobacteria, *Campylobacter lanie*, and *Desulfovibrio piger*. This could indicate that the interaction between inulin and *T. suis* may modulate the microbiota, diverting it from a potentially inflammatory state in some parts of the intestine. This is further supported by our recent paper, in which we observed a synergistically activated Th2 response and a suppressed Th1 response; indicated by up-regulation of *IL13* and *TFF3* and down-regulation of pro-inflammatory genes such as *IFNG* and *CXCL9* in the interaction group (Myhill et al., 2018).

The decrease in *Desulfovibrio* following *T. suis* infection might also influence epithelial mucin production. If *Desulfovibrio* is found in co-occurrence with *Prevotella* species, mucin degradation is increased by hydrolyzing enzymes generated by *Prevotella*, which cannot be activated before desulfation by *Desulfovibrio* (Wright et al., 2000). We noticed a decrease in *Desulfovibrio* across all experimental groups in the present study, which could indicate a reduced degradation of mucins. In our recent paper, we found an increased number of mucin-producing goblet cells in *T. suis*-infected pigs compared with uninfected pigs (Myhill et al., 2018) resulting in a thickening of the mucus layer. The current decrease in *Desulfovibrio* might therefore be a part of the host’s “weep and sweep” response to expel *T. suis* (Anthony et al., 2007).

We observed only a minor, non-significant reduction in worm burden in the inulin-supplemented group, accompanied by less variation between worm burdens compared with the standard diet. Earlier studies utilizing inulin as an anthelmintic treatment showed differing results, ranging from increased worm burdens in pigs fed chicory (Jensen et al., 2011) to reduced *T. suis* burdens when a higher level of commercial inulin was used (Petkevicius et al., 2006). However, in pigs, the effect of inulin supplementation on the GM varies greatly depending on the dose and chain length of inulin, age of the pig and sampling location (Metzler-Zebeli et al., 2017), and any direct effect of inulin on *T. suis* might also be dependent on these factors. The study was terminated at day 28 p.i., when the *T. suis* were still immature, and any direct and/or indirect effects of inulin may be more pronounced at later stages.

Taken together, our present findings and also the associated immunological findings (Myhill et al., 2018) add to a growing body of research, that suggests that helminth infection might to some degree be beneficial to its host, e.g. through reduction of excessive inflammation and thus potentially, alleviation of allergic or autoimmune diseases in humans (Giacomin et al., 2015; Loke and Lim, 2016). A lower relative abundance of Proteobacteria was observed for *T. suis* infected groups, minimizing the colonization of potential bacterial pathogens in the pig’s intestine. The higher relative abundance of *Bifidobacterium*, *Prevotella*, *Lactobacillus*, and other species associated with gut health found in the combination group indicates that the inulin and *T. suis* may interact to improve the gut health of pigs. This may be manifested by either a direct effect of altered immune responses following an infection, or an indirect interaction with the microbiota that modulates inflammation and immune function.

The gut health of a pig is mostly focused on the absence of gastrointestinal-related diseases, and it has earlier been proposed that the focus should be on an optimal microbiota rather than a normal microbiota. An optimal GM composition is able to maintain an equilibrium despite gastrointestinal challenges such as bacterial pathogens or parasites, which otherwise would result in a rapid population shift (Pluske et al., 2018). Diet is one of the major factors affecting microbial composition, and alterations in macronutrients will affect the composition (Aluthge et al., 2019). Additionally, prebiotics such as inulin with perceived health benefits have the potential to directly alter the microbiota through proliferation of the already resident beneficial bacteria (Nowland et al., 2019). In our study, we saw that inulin supplementation results in a specific microbial composition which persists over the course of 4 weeks. As mentioned earlier, the introduction of *T. suis*, which normally would be regarded as a gastrointestinal challenge, did not revert the positive effects seen from inulin supplementation but enhanced them. However, it is important to note that the composition of feed changes dependent of life stage of the pig, and the microbial alterations due to inulin might be less evident at earlier stages. Likewise, one should be careful to introduce inulin (and *T. suis*) at a too high percentage, as it could be detrimental for the pig. Naturally, there is a need for more studies investigating potential health promoting factors resulting from the interaction between inulin and *T. suis*.

Hence, both *T. suis* and inulin and their interaction drive changes in GM, which can be putatively characterized as positive, in the sense that the interaction lowers the abundance of dysbiosis-related taxa while increasing the abundance of species indicative of a healthy gut.

## Data Availability Statement

The datasets generated for this study can be found in the European Nucleotide Archive (ENA); accession number PRJEB29079.

## Ethics Statement

This study was conducted in line with the Danish Animal Experimentation Inspectorate (License No: 2015-15-0201-00760) and approved by the Experimental Animal Unit, University of Copenhagen according to FELASA guidelines and recommendations.

## Author Contributions

AW, ST, HM, PN, LM, and SS conceived the project and experiments. LM, SS, AW, ST, HM, and PN performed the animal study. SS performed all laboratory analyses. CS and LA guided 16S rRNA sequencing experimental design, samples processing, and data analysis. LK and DN guided 16S rRNA data analysis. SS and ST prepared the manuscript with input from all other authors. All authors reviewed the final manuscript.

## Conflict of Interest

The authors declare that the research was conducted in the absence of any commercial or financial relationships that could be construed as a potential conflict of interest.
