# Supplementary material for: Dietary Inulin and Trichuris suis Infection Promote Beneficial Bacteria Throughout the Porcine Gut
Source: Front Microbiol. 2020 Mar 4;11:312. doi: 10.3389/fmicb.2020.00312 (PMC7064446; doi:10.3389/fmicb.2020.00312)

***Table S1*: Composition of standard (control) and experimental inulin-supplemented diet.** Both diets were pre-mixed, and balanced for crude protein and energy content. Purified long-chain inulin contained average DP ≥ 23.

Ingredient (g/kg)	Standard	Inulin
Barley	577	293
Wheat	163	290
Inulin[1]	-	100
Soybean meal	202	228
Palm oil distillate fatty acids	15	30
Molasses	15	12
Calcium carbonate	13	13
Calcium phosphate	7	7
Sodium chloride	3	4
L-lysine hydrochloride	2	2
Methionine	1	1
Vitamin mix	2	2
**Calculated composition**		
Crude protein (g/kg)	160	160
Metabolisable Energy (MJ/kg)	12.59	12.47
**Analysed composition**		
Dry matter (%)	90.7	91.1
Fat (%)	4.1	5.1
Crude Fibre (%)	3	3
Ash (%)	5.2	5.1
Lysine (%)	0.97	0.95
Methionine (%)	0.27	0.27
Calcium (%)	0.74	0.73
Phosphorous (%)	0.48	0.46
Sodium (%)	0.16	0.17
Crude protein (g/kg)	162	173
Metabolisable Energy (MJ/kg)	13.94	14.53

Figure S1: **Experimental set-up. (A)** A 2-factorial study design illustrates dietary and infection treatment groups. **(B)** Time line of the study.

A

Diet \ Infection	Naïve	*T. suis* infection
Basal diet	**Control** (8 pigs)	***T. suis*** (9 pigs)
10% Inulin supplementation	**Inulin** (8 pigs)	**Inulin+ *T.suis*** (9 pigs)

B

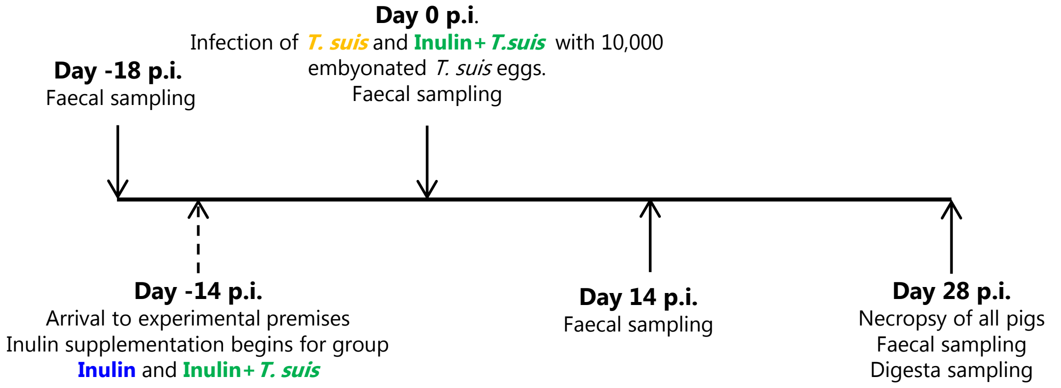

***Table S2:* Primer combinations used for PCR1 targeting V3-V4 of the 16S region.** Forward (Fwd) Primer (red) and Fwd inserts (of 5-17 nt length) (green), and reverse (Rev) primer (blue) and Rev inserts (of 6-20 nt length) (orange).

	Fwd insert	*Fwd* primer
		ACTCCTAYGGGRBGCASCAG
i5	CGCAG	ACTCCTAYGGGRBGCASCAG
i16	GTGTAGTAATCTGAAT	ACTCCTAYGGGRBGCASCAG
i17	TAAGTCGGTAACACTCT	ACTCCTAYGGGRBGCASCAG
	***Rev* insert**	***Rev* primer**
		AGCGTGGACTACNNGGGTATCTAAT
r6	GATCAT	AGCGTGGACTACNNGGGTATCTAAT
r12	CTGAGCCTGCG	AGCGTGGACTACNNGGGTATCTAAT
r20	TCATCATCAACTCTACT	AGCGTGGACTACNNGGGTATCTAAT

Figure S2: **Number of species present in each treatment group** at **(A)** each study time point, and **(B)** in each gut segment at day 28 post-infection (p.i.) Data are represented as means with minimum and maximum values, and error bars represent SD . (Group C = control; Group Ts = *Trichuris suis*; Group I = Inulin; Group I+Ts = Inulin+*T. suis*. Il = ileum; Ca = caecum; PC = proximal colon; DC = distal colon).

A

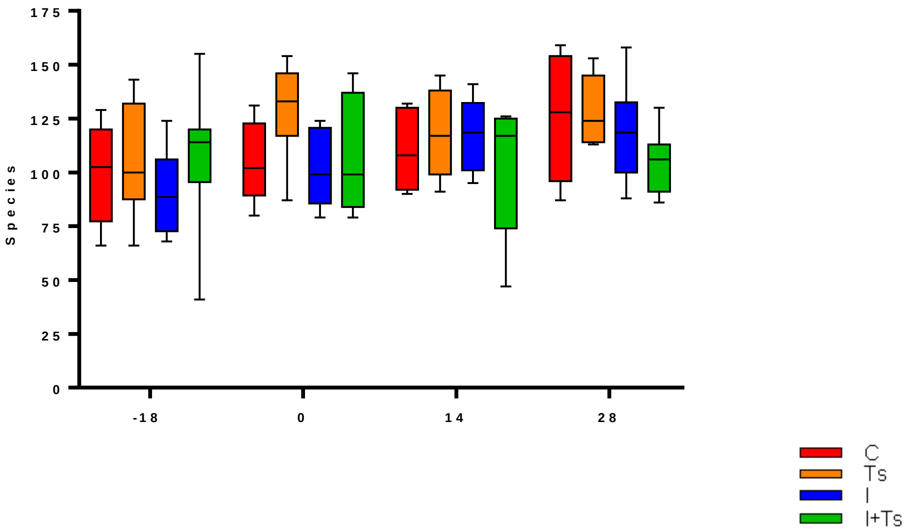

B

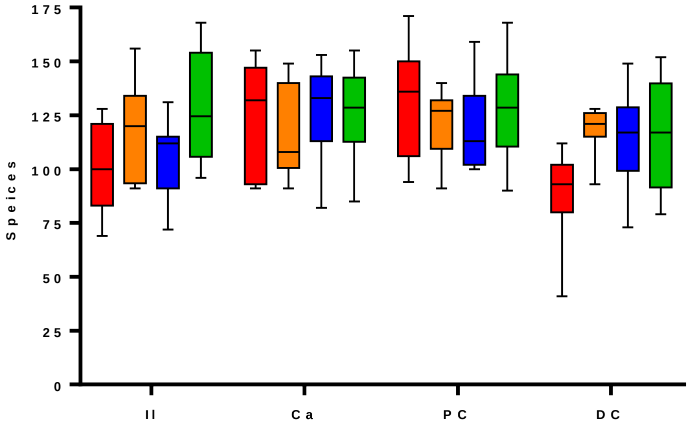

***Table S3:* PERMANOVA *p*-values for Sorensen-Dice (S-D) and Bray Curtis (BC) for pair-wise comparisons at** (**A**) between groups as each time point (**B**) between groups for each intestinal segment (**C**) between two time points within each group (**D**) between two intestinal segments within each group. *p*-value < 0.05 is considered significant between the two groups.

A

			Day -18 p.i.		Day 0 p.i.		Day 14 p.i.		Day 28 p.i.	
			S-D	BC	S-D	BC	S-D	BC	S-D	BC
Control	vs.	*T. suis*	0.680	0.819	0.056	0.030	0.071	0.162	0.028	0.093
Control	vs.	Inulin	0.282	0.236	0.002	0.002	0.006	0.019	0.002	0.007
Control	vs.	Inulin+ *T.suis*	0.456	0.287	0.002	0.002	0.002	0.001	0.001	0.001
T. suis	vs.	Inulin	0.490	0.160	0.001	0.004	0.011	0.023	0.002	0.002
T. suis	vs.	Inulin+ *T.suis*	0.324	0.215	0.005	0.005	0.001	0.006	0.001	0.001
Inulin	vs.	Inulin+ *T.suis*	0.008	0.029	0.580	0.725	0.190	0.418	0.005	0.037

B

			Ileum		Caecum		Proximal Colon		Distal Colon	
			S-D	BC	S-D	BC	S-D	BC	S-D	BC
Control	vs.	*T. suis*	0.001	0.031	0.053	0.069	0.020	0.045	0.004	0.066
Control	vs.	Inulin	0.003	0.002	0.002	0.003	0.002	0.003	0.010	0.016
Control	vs.	Inulin+ *T.suis*	0.001	0.001	0.001	0.002	0.001	0.002	0.001	0.003
T. suis	vs.	Inulin	0.001	0.008	0.001	0.002	0.001	0.016	0.002	0.017
T. suis	vs.	Inulin+ *T.suis*	0.001	0.001	0.001	0.003	0.001	0.010	0.001	0.001
Inulin	vs.	Inulin+ *T.suis*	0.001	0.043	0.081	0.144	0.491	0.779	0.536	0.446

C

			**Control**		***T. suis***		**Inulin**		**Inulin+ *T.suis***	
			S-D	BC	S-D	BC	S-D	BC	S-D	BC
Day -18 p.i.	Vs.	Day 0 p.i.	0.001	0.005	0.001	0.001	0.001	0.001	0.001	0.001
Day -18 p.i.	Vs.	Day 14 p.i.	0.002	0.001	0.001	0.001	0.001	0.011	0.001	0.001
Day -18 p.i.	Vs.	Day 28 p.i.	0.002	0.035	0.001	0.002	0.001	0.001	0.001	0.001
Day 0 p.i.	Vs.	Day 14 p.i.	0.091	0.014	0.058	0.330	0.869	0.370	0.005	0.111
Day 0 p.i.	Vs.	Day 28 p.i.	0.061	0.055	0.004	0.197	0.065	0.349	0.001	0.026
Day 14 p.i.	Vs.	Day 28 p.i.	0.426	0.015	0.069	0.190	0.075	0.249	0.014	0.046

D

			**Control**		***T. suis***		**Inulin**		**Inulin+ *T.suis***	
			S-D	BC	S-D	BC	S-D	BC	S-D	BC
Ileum	Vs.	Caecum	0.001	0.002	0.001	0.001	0.002	0.002	0.001	0.001
Ileum	Vs.	Proximal colon	0.002	0.001	0.001	0.001	0.002	0.001	0.001	0.001
Ileum	Vs.	Distal colon	0.001	0.001	0.001	0.001	0.002	0.002	0.001	0.001
Caecum	Vs.	Proximal colon	0.982	0.814	0.894	0.996	0.930	0.579	0.942	0.665
Caecum	Vs.	Distal colon	0.005	0.026	0.226	0.086	0.178	0.021	0.001	0.039
Proximal colon	Vs.	Distal colon	0.014	0.04	0.077	0.117	0.593	0.261	0.009	0.063

***Table S4: p*- and R values for pair-wise group comparisons at the four study time points.** (**A**) Sorensen-Dice (**B**) Bray Curtis. *p*-value < 0.05 is considered significant between the two groups. R-value > 0.5 indicates dissimilarities between the two groups.

A

			Day -18 p.i.		Day 0 p.i.		Day 14 p.i.		Day 28 p.i.	
			P value	*R value*	*P value*	*R value*	*P value*	*R value*	*P value*	*R value*
Control	vs.	*T. suis*	0.671	0.000	0.021	0.174	0.110	0.097	0.037	0.183
Control	vs.	Inulin	0.298	0.033	0.002	0.431	0.021	0.186	0.002	0.458
Control	vs.	Inulin+ *T.suis*	0.865	0.000	0.002	0.353	0.001	0.401	0.001	0.899
T. suis	vs.	Inulin	0.533	0.000	0.001	0.466	0.006	0.237	0.001	0.529
T. suis	vs.	Inulin+ *T.suis*	0.671	0.000	0.006	0.224	0.001	0.463	0.001	0.765
Inulin	vs.	Inulin+ *T.suis*	0.016	0.228	0.632	0.000	0.215	0.053	0.005	0.322

B

			Day -18 p.i.		Day 0 p.i.		Day 14 p.i.		Day 28 p.i.	
			P value	*R value*	*P value*	*R value*	*P value*	*R value*	*P value*	*R value*
Control	vs.	*T. suis*	0.889	0.000	0.035	0.233	0.261	0.040	0.106	0.122
Control	vs.	Inulin	0.262	0.029	0.002	0.486	0.031	0.217	0.003	0.461
Control	vs.	Inulin+ *T.suis*	0.352	0.000	0.002	0.529	0.001	0.518	0.001	0.624
T. suis	vs.	Inulin	0.220	0.050	0.003	0.370	0.016	0.266	0.002	0.646
T. suis	vs.	Inulin+ *T.suis*	0.427	0.000	0.005	0.319	0.004	0.395	0.001	0.578
Inulin	vs.	Inulin+ *T.suis*	0.029	0.329	0.786	0.000	0.3815	0.002	0.095	0.156

***Table S5: p*- and R values for time point comparisons within each group.** (**A**) Sorensen-Dice index (**B**) Bray Curtis index. *p*-value < 0.05 is considered significant between the two time points. R-value > 0.5 indicates dissimilarities between the two groups. (C = control; Ts = *Trichuris suis*; I = Inulin; I+Ts = Inulin +*T. suis*).

A

			Control		*T. suis*		Inulin		Inulin+ *T.suis*	
			P value	*R value*	*P value*	*R value*	*P value*	*R value*	*P value*	*R value*
Day -18 p.i.	Vs.	Day 0 p.i.	0.002	0.292	0.001	0.379	0.001	0.710	0.001	0.394
Day -18 p.i.	Vs.	Day 14 p.i.	0.003	0.332	0.001	0.438	0.001	0.558	0.001	0.421
Day -18 p.i.	Vs.	Day 28 p.i.	0.003	0.353	0.001	0.568	0.001	0.615	0.001	0.783
Day 0 p.i.	Vs.	Day 14 p.i.	0.203	0.054	0.082	0.082	0.925	0.000	0.011	0.190
Day 0 p.i.	Vs.	Day 28 p.i.	0.183	0.068	0.004	0.232	0.116	0.093	0.002	0.427
Day 14 p.i.	Vs.	Day 28 p.i.	0.319	0030	0.097	0.081	0.264	0.037	0.009	0.138

B

			Control		*T. suis*		Inulin		Inulin+ *T.suis*	
			P value	*R value*	*P value*	*R value*	*P value*	*R value*	*P value*	*R value*
Day -18 p.i.	Vs.	Day 0 p.i.	0.005	0.452	0.001	0.477	0.001	0.626	0.001	0.544
Day -18 p.i.	Vs.	Day 14 p.i.	0.002	0.589	0.001	0.515	0.011	0.377	0.001	0.570
Day -18 p.i.	Vs.	Day 28 p.i.	0.027	0.289	0.002	0.400	0.001	0.533	0.001	0.748
Day 0 p.i.	Vs.	Day 14 p.i.	0.018	0.379	0.282	0.027	0.393	0.000	0.104	0.117
Day 0 p.i.	Vs.	Day 28 p.i.	0.049	0.228	0.235	0.033	0.195	0.072	0.057	0.157
Day 14 p.i.	Vs.	Day 28 p.i.	0.017	0.265	0.241	0.040	0.124	0.107	0.057	0.160

***Table S6: p*- and R values for pair-wise group comparisons at the four intestinal locations.** (**A**) Sorensen-Dice (**B**) Bray Curtis. *p*-value < 0.05 is considered significant between the two groups. R-value > 0.5 indicates dissimilarities between the two groups.

A

			Ileum		Caecum		Proximal colon		Distal colon	
			P value	*R value*	*P value*	*R value*	*P value*	*R value*	*P value*	*R value*
Control	Vs.	*T. suis*	0.002	0.422	0.031	0.173	0.015	0.227	0.002	0.450
Control	Vs.	Inulin	0.008	0.284	0.001	0.357	0.001	0.454	0.009	0.543
Control	Vs.	Inulin+ *T.suis*	0.001	0.664	0.001	0.647	0.001	0.586	0.001	0.841
T. suis	Vs.	Inulin	0.001	0.557	0.003	0.335	0.001	0.516	0.002	0.591
T. suis	Vs.	Inulin+ *T.suis*	0.002	0.747	0.001	0.611	0.001	0.531	0.001	0.702
Inulin	Vs.	Inulin+ *T.suis*	0.002	0.317	0.077	0.128	0.538	0.000	0.413	0.004

B

			Ileum		Caecum		Proximal colon		Distal colon	
			P value	*R value*	*P value*	*R value*	*P value*	*R value*	*P value*	*R value*
Control	Vs.	*T. suis*	0.041	0.201	0.130	0.094	0.055	0.167	0.043	0.250
Control	Vs.	Inulin	0.002	0.503	0.003	0.458	0.003	0.391	0.013	0.490
Control	Vs.	Inulin+ *T.suis*	0.001	0.828	0.003	0.461	0.002	0.478	0.002	0.736
T. suis	Vs.	Inulin	0.007	0.414	0.003	0.343	0.008	0.309	0.005	0.393
T. suis	Vs.	Inulin+ *T.suis*	0.001	0.664	0.003	0.443	0.006	0.298	0.001	0.525
Inulin	Vs.	Inulin+ *T.suis*	0.044	0.145	0.154	0.087	0.686	0.000	0.371	0.014

***Table S7: p*- and R values for pair-wise comparisons within each group for gut segments.** (**A**) Sorensen-Dice index (**B**) Bray Curtis index. *p*-value < 0.05 is considered significant between the two groups. R-value > 0.5 indicates dissimilarities between the two segments.

A

			Control		T. suis		Inulin		Inulin+ T.suis	
			P value	*R value*	*P value*	*R value*	*P value*	*R value*	*P value*	*R value*
Ileum	Vs.	Caecum	0.002	0.826	0.001	0.658	0.002	0.667	0.001	0.802
Ileum	Vs.	Proximal colon	0.002	0.867	0.001	0.708	0.002	0.784	0.001	0.881
Ileum	Vs.	Distal colon	0.002	0.899	0.001	0.819	0.003	0.605	0.001	1.000
Caecum	Vs.	Proximal colon	0.942	0.000	0.879	0.000	0.963	0.000	0.873	0.000
Caecum	Vs.	Distal colon	0.009	0.244	0.224	0.039	0.105	0.090	0.001	0.359
Proximal colon	Vs.	Distal colon	0.017	0.257	0.066	0.103	0.770	0.000	0.006	0.230

B

			Control		T. suis		Inulin		Inulin+ T.suis	
			P value	*R value*	*P value*	*R value*	*P value*	*R value*	*P value*	*R value*
Ileum	Vs.	Caecum	0.001	0.843	0.001	0.725	0.001	0.810	0.001	0.900
Ileum	Vs.	Proximal colon	0.002	0.812	0.001	0.784	0.002	0.747	0.001	0.876
Ileum	Vs.	Distal colon	0.001	0.953	0.001	0.788	0.002	0.564	0.001	0.964
Caecum	Vs.	Proximal colon	0.811	0.000	0.998	0.000	0.460	0.000	0.697	0.000
Caecum	Vs.	Distal colon	0.023	0.312	0.078	0.117	0.013	0.209	0.035	0.223
Proximal colon	Vs.	Distal colon	0.042	0.259	0.115	0.084	0.168	0.077	0.050	0.189

***Figure S3:* Heatmap of significant differentially abundant taxa between the four treatment groups found by ANCOM at four time points.** Heatmap of significant different log abundance of faecal taxa at day -18 post-infection (p.i.), day 0 p.i., day 14 p.i. and day 28 p.i. Only taxa found to be significantly different by ANCOM (*p*<0.05) between two or more groups are included. X denotes no difference in abundance in the segment, not non-presence. (C = control; Ts = *Trichuris suis*; I = Inulin; I+Ts = Inulin +*T. suis*).

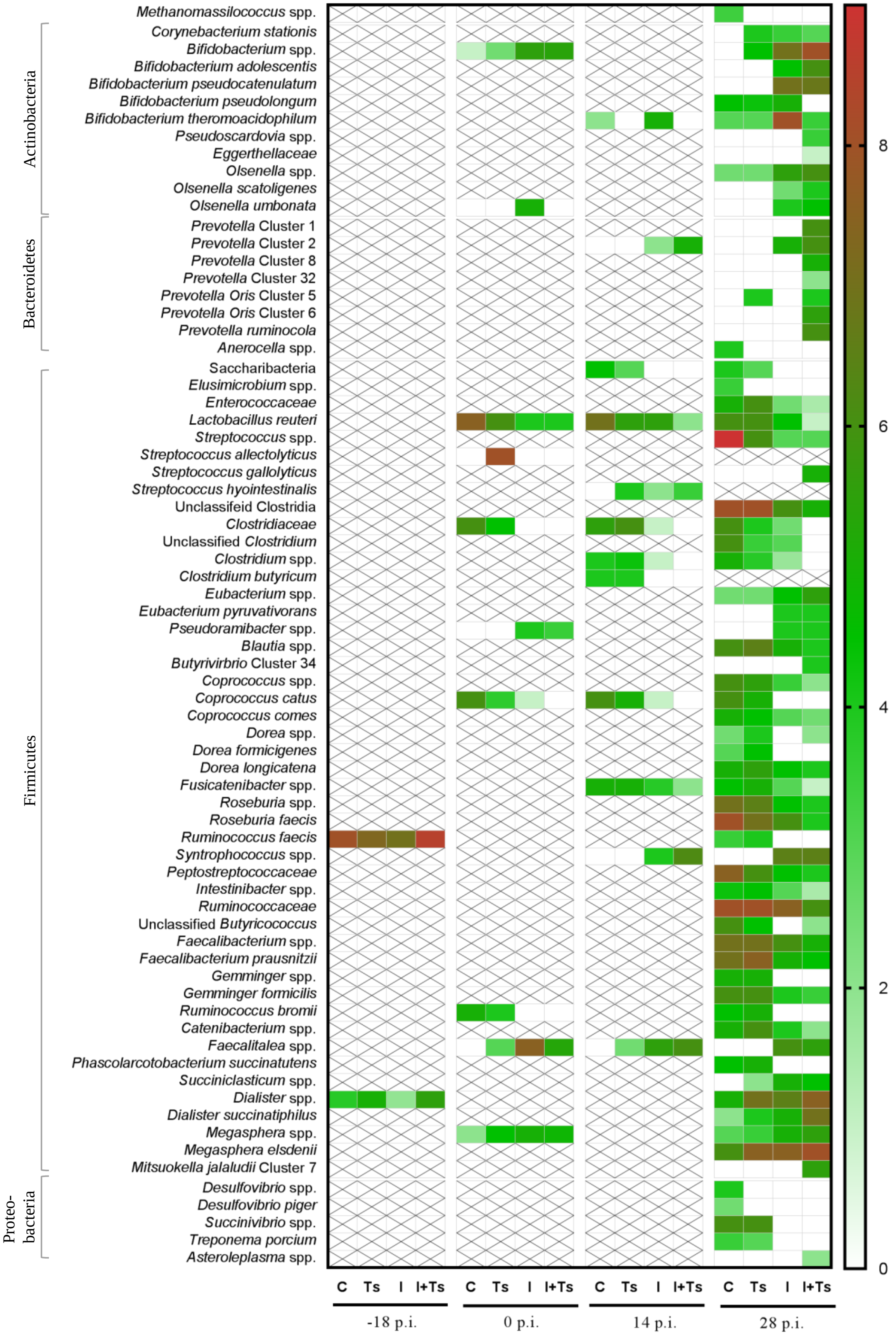

Supplement: Supplementary file 1 [file Data_Sheet_1.PDF]
